# Implementation of evidence-based rehabilitation for non-specific back pain and common mental health problems: a process evaluation of a nationwide initiative

**DOI:** 10.1186/s12913-015-0740-4

**Published:** 2015-02-28

**Authors:** Elisabeth Björk Brämberg, Charlotte Klinga, Irene Jensen, Hillevi Busch, Gunnar Bergström, Mats Brommels, Johan Hansson

**Affiliations:** Division of Intervention and Implementation Research (IIR), Institute of Environmental Medicine (IMM), Karolinska Institute, SE 171 77 Stockholm, Sweden; Department of Learning, Informatics, Management and Ethics (LIME), Medical Management Centre (MMC), Karolinska Institute, SE 171 77 Stockholm, Sweden; The National Board of Health and Welfare, SE 106 30 Stockholm, Sweden; Centre for Occupational and Environmental Medicine, Stockholm County Council, Stockholm, Sweden

**Keywords:** Back pain, Common mental health problems, Evidence-based, Implementation, Process evaluation

## Abstract

**Background:**

Nationwide implementation of guaranteed access to evidence-based rehabilitation was established in Sweden in 2009, through an Act of the Swedish Government. The rehabilitation guarantee’s primary goal was to increase the rate of return-to-work, reduce and prevent long-term absenteeism after diagnoses related to back pain and common mental health problems. This study aims to develop knowledge about factors influencing large-scale implementation of complex and extensive interventions in healthcare settings.

**Methods:**

Three different data sources questionnaires, interviews and documents were used in data collection and analysis. The data were analysed using iterative thematic analysis.

**Results:**

The following main facilitators contributed to realization of the rehabilitation guarantee: financial incentives, establishment of project organization, recruitment, in-service training and previous experiences of working in similar projects. Barriers were: the rehabilitation guarantee’s short-term project-form, clinicians’ attitudes to and competence in working towards return-to-work, lack of guidelines describing treatment modalities in multimodal rehabilitation, and lack of well-defined criteria for inclusion of patients. Documents revealed that the return-to-work goal became less pronounced during the implementation process. Instead, care and health were more often described in documents used to disseminate information about the rehabilitation guarantee. Intermediate outcomes found were: patients with rehabilitation needs were given more adequate priority, increased readiness for future implementation efforts, and increased general competence in psychotherapy, and team-work, which thus became available to patient groups other than those covered by the rehabilitation guarantee.

**Conclusions:**

To facilitate implementation of established national policy goals in clinical practice, tools are needed that specifically aim at changing clinicians’ attitudes and behaviours in relation to such goals. Our results underline the importance of investing both time and sufficient resources in the activities and in supporting the implementation process.

## Background

### Diagnoses and evidence-based rehabilitation

The lifetime prevalence of back, neck, and shoulder pain is estimated to be 70–80% in the general population [1]. These conditions are the greatest causes of work incapacity in Europe [2,3]. The first three months after pain onset is crucial to recovery. Persons who do not spontaneously recover within this period are at risk of transiting from acute to persistent back pain [4]. Back pain is primarily nonspecific in origin; specific medical causes are found in less than 15% of cases [5].

Multimodal rehabilitation (MMR) is the rehabilitation method of choice in international guidelines for back pain treatment. There is evidence that MMR reduces symptoms, disability, and absenteeism [6], that it is cost-effective [7] up to ten years after the intervention [8,9].

MMR is an intensive form of rehabilitation with a biopsychosocial perspective, reflected in a multiprofessional team, usually involving at least a physician, a psychologist, and a physiotherapist and/or an occupational therapist. MMR is given on a full- or part-time basis, for about 2 to 8 weeks. Central objectives are enhancing the individual’s understanding of the mechanisms and consequences of pain as well as teaching methods for managing pain in everyday life (including working life). MMR programmes often consist of education, exercise therapy, and cognitive behavioural treatment [8,10].

The prevalence of mental health problems related to anxiety, depression, and stress-related disorders is estimated to be about one-third of the European population (between the ages of 18–65 years), and has increased during the period 2005 to 2010 [11,12]. Work absence due to mental health problems has become more common than absence due to musculoskeletal diseases, which until recently constituted the most common cause in Sweden [13].

Few studies have evaluated therapies addressing common mental health disorders in relation to return-to-work outcomes. Psychotherapeutic interventions such as cognitive behavioural therapy, cognitive therapy, and interpersonal psychotherapy have been shown to reduce symptoms related to anxiety [14] and light to moderate depression [15].

### The Swedish healthcare context and the rehabilitation guarantee

The Swedish healthcare system is organized on three independent government levels: the national level, the county council level (21 county councils) and the municipal level (290 municipalities). At the national level, the Ministry of Health and Social Affairs establishes overarching principles and guidelines for health and social care. The main responsibility for provision of healthcare services lies with the county councils and regions. At the most local level, the municipalities are responsible for social welfare services and care delivered in people’s homes [16].

Nationwide implementation of a rehabilitation guarantee (RG) was established in Sweden in 2009. The starting point was a political resolution passed by the Swedish Government, in line with the Government’s work-first principle, i.e. measures to increase establishment on the labour market, and to increase return-to-work for those absent from work due to illness. The Government introduced the RG policy to address the latter issue, aimed at persons suffering from non-specific back pain and common mental health problems, by means of increased accessibility to evidence-based rehabilitation for certain diagnoses. About 10% of all persons on sick leave were affected by the policy. Diagnoses related to mental disorders and musculoskeletal system diseases are the most common causes of long-term illness in Sweden, accounting for 68% of public health insurance costs [13].

The aims of the RG’s are to increase return-to-work and prevent long-term absenteeism for diagnoses related to back pain and common mental health problems by increasing accessibility to evidence-based therapies. The core components of the RG were: MMR for back pain treatment and cognitive behavioural therapy, cognitive therapy and interpersonal psychotherapy for treatment of common mental health problems. Below, cognitive behavioural therapy, cognitive therapy and interpersonal psychotherapy are collectively referred to as psychotherapeutic techniques (PT). The RG comprises all individuals of working age, i.e., 16–67 years of age, who are at risk for long-term absenteeism. Since its start in 2009, the RG is run on a yearly basis with annual renegotiations taking place between stakeholders. To stimulate implementation of the RG, the Government allocated 250 million SEK during 2008 and agreed to pay the county councils 15,000 SEK per patient in psychotherapy and 45,000 SEK per patient in MMR during the first years of the RG. The Swedish Association of Local Authorities and Regions is a member organization for municipalities, county councils and regions; its mission is to enhance members’ possibilities to provide conditions for local and regional self-government. The Swedish Association of Local Authorities and Regions’s role in the RG was to negotiate the RG with the Swedish Government and to support implementation by providing the county councils with information on the RG, educating process managers, and providing guidelines and organization for workshops. From the start of the RG, the Government allocated funds for evaluating the effects of the RG. These evaluations were commissioned to universities and other government agencies. Authorities were not involved in the decisions or the implementation of the RG.

Implementation of the RG within the county councils was not required by the Government. Decisions concerning implementation strategies were left to the county councils, the aim being to facilitate adaption to the local context and conditions. To participate in the RG, the county councils were required to offer at least one of the treatments included in the RG (i.e., MMR and PT) and to report completed treatments to the Swedish Association of Local Authorities and Regions. The implementation strategy was top-down only in that it defined which treatments to offer to specific patients. Adherence to the governmental system for reporting completed treatments to receive financial support was mandatory. The 21 county councils in Sweden were invited by the Government to participate in the RG. All 21 accepted the invitation. Each county council assigned a process manager who had overall responsibility for implementation of the RG. Besides these common features, the individual counties’ prerequisites varied greatly, at the outset in 2009 and throughout the implementation process, regarding staffing levels, previous experience of similar initiatives and current service conditions.

Implementation of the RG is being studied from different perspectives. The present study addresses the implementation process. A controlled effectiveness study of the RG’s outcomes on the patient level was also performed. The controlled effectiveness study employed a matched cohort design. The results showed a positive effect favouring the intervention group (the RG group), with a significant increase in health and self-reported work ability, a significantly lower proportion of disability pension, but no effect on sick leave (manuscript submitted for international publication) [17,18]. It further showed that RG was cost effective compared to the typical treatment, mainly due to its effect on reducing the risk of disability pension.

### Implementation of complex interventions

Authorities worldwide have taken measures to improve the quality and efficiency of health and social care, with varying degrees of success [19-21], and many theories and models have been developed to explain how changes can be made in health and social care [22]. As a means of improving care, a simple passive diffusion of information approach has proven insufficient in bringing about efficient change [23].

Implementing complex and extensive interventions, such as the RG, is an intricate and challenging process that requires special attention to factors that facilitates and hinder implementation. There is a well-recognized need for a holistic case-study approach addressing the content of the intervention, how the actions taken to implement change are received and developed, the intervention context, and intervention outcomes [24,25]. Walshe [26] stressed the need to unpack the complex relationship between context, content, application, and outcomes to better understand when, how, and why an intervention works.

The success of implementing methods is dependent on contextual factors that influence the intervention’s outcome(s) and sustainability. Examples of facilitating factors are establishment of the intervention on the management level (legitimacy), leadership, contribution to organizational change, staff resources and willingness to try new processes [27-29]. Potential barriers are parallel development activities, competing agendas, demands [28], and resistance to change in the organization and surrounding context [29]. Factors may influence change on different levels: (1) the individual care provider level (e.g., a provider’s competence, motivation for change, and individual characteristics), (2) the social setting level (e.g., the patient’s age, sex, and socioeconomic status), and (3) the system level (e.g., organization and financial resources) [30].

### Theoretical perspective

The present study uses the model of strategic change management developed by Pettigrew and Whipp [31], which is frequently used in analysing change programmes in organizations [32,33]. The model focuses on the content of the change, how the actions taken to implement the change are received and developed, and the context, along with intermediate and final outcomes [26,32,34]. Implementation scientists have called for studies that highlight contextual issues [27,33]. Attempts to standardize interventions, to make them - “one size fits all” - risk missing the point that the outcomes of large-scale interventions are interrelated with context, content, and implementation actions [26].

Thus, the present study aims to develop knowledge about factors influencing large-scale implementation of complex and extensive interventions in healthcare settings. To achieve this, we explore nationwide implementation of evidence-based rehabilitation for back pain and common mental health problems.

The following research questions are addressed:

- How was the implementation organized from the national to the clinical level?
- What were the contextual factors influencing the implementation?
- What were the intermediate outcomes of the RG’s intentions?

## Method

The study was part of an evaluation commissioned by the Ministry of Health and Social Affairs. An assessment of the RG’s effectiveness has been reported elsewhere (international manuscript submitted for publication) [17,18]. The current study focused on the implementation process. The research group had an independent and external role with no impact on the implementation process, the content or outcome of the RG. The study has a process evaluation design using a method triangulation approach that combines data from questionnaires, interviews, and documents. As these sources addressed slightly different facts we choose to use the information for complementary purposes rather than for convergence of evidence. Nevertheless, triangulation is useful for understanding how the context may influence the implementation process and for providing insights to aid future implementation in dynamic and complex settings [35].

Based on this theoretical framework, data reflecting the following themes were collected: the content of the RG (the ‘what’ of strategic change, i.e., objectives and assumptions); the context (the healthcare unit environment in which changes take place, characterized by, e.g., organizational culture, politics, leadership, and clinical settings); the process of implementing the RG (the ‘how’ of strategic change, i.e., the methods of change and implementation strategies); and intermediate outcomes.

### Selection of county councils and participants

A questionnaire was developed that contained three main themes: opinions on the RG (‘To what extent do you consider that the agreement for realization of the RG was clearly stated?’), implementation of the RG (‘Did the county council develop written plans/action plans for the implementation?’), and changes related to the implementation process (‘Did the realization result in recruitment?’). The response formats consisted of Likert-type scales or were open-ended. The Swedish Association of Local Authorities and Regions provided the research team with a list of 118 e-mail addresses for healthcare directors, process managers, and first-line managers in healthcare units in 21 county councils in Sweden. The response rate was 59% at the healthcare units and 61% at the administration level, giving an average of 60%.

Based on the questionnaire results, six county councils were selected for interviews and document reviews, the aim of selection being to maximize case variation [36]. The selection was guided by the responses to four key questions from the questionnaire. These questions addressed (a) the observed clarity of the description of the RG, (b) access to written plans for implementation of the RG, (c) the perceived attitudes among employees/colleagues towards working in accordance with the RG, and (d) the perceived priority of implementing the RG in practice. A 5-point scale ranging from “totally disagree” to “totally agree” was used. Counties with a preponderance of positive (n = 3) and/or negative (n = 3) answers were selected.

The selection of participants for the interviews was guided by strategic sampling, i.e. interviewees should represent different modalities of healthcare. For each county council, we recruited persons from the management level who were responsible for implementation, including healthcare directors and process managers. Furthermore, first-line managers from the healthcare sector representing specialized care or primary care (responsible for delivering RG), as well as both kinds of ownership, i.e. public or private were included.

In the county council management, the research assistant (CK) identified and contacted process managers. Thereafter, the process managers identified possible participants (i.e., healthcare directors and first-line managers or equivalent). The process managers briefly informed about the study’s aim and assigned healthcare directors and first-line managers (or equivalent) to an interview.

Process managers and first-line managers at healthcare units in the six county councils were asked to send the documents that they were using, either at the time of the inquiry or previously, concerning the RG. A reminder was sent by e-mail to those who had not responded within two weeks. All counties sent documents, resulting in a total of 165 documents.

### Data collection

#### Questionnaire

From the questionnaire, responses to the open-ended questions are used as data. The questions were: ‘What circumstances/factors have, until today, facilitated the council’s implementation of the RG? Mention the three most common circumstances/factors.’; ‘What circumstances/factors have, until today, been potential barriers to the council’s implementation of the RG? Mention the three most common potential barriers’.

#### Interviews

Semi-structured interviews with open-ended questions were conducted face-to-face with 34 participants at their workplaces. A total of 36 subjects were invited; 2 persons declined participation. The reasons given were lack of time or illness. First-line managers from privately owned care facilities were represented to a lesser degree than were first-line managers from publicly run care facilities.

The interview guide was pilot-tested and minor revisions were made after the interview. The pilot interview was not included in the data. The final interview guide addressed: the aim and realization of the RG, documents related to the RG, structures for communication and decisions related to the RG, facilitators, and possible barriers and changes related to implementation of the RG. Follow-up questions were also asked (e.g., ‘Can you tell me more about that?’).

All interviews were conducted in May 2011 by the second author (CK). Data collection was completed approximately two years after the official launch of the national programme. Due to local requirements the starting point of the RG varied across the county councils. At the time of data collection, the RG outcome as revealed in the effectiveness study [18] was known to the public. All participants had been employed at their current positions since the implementation started. The interviews lasted between 50 and 70 minutes and were digitally recorded and transcribed verbatim. No compensation was paid.

#### Document review

A screening of the submitted documents from the county councils was made to identify relevant documents. Documents such as guidelines for using computer programmes when reporting patients’ therapies, or forms to be completed by patients or staff, were excluded. Of the165 documents, 85 documents were included in the analysis.

### Data analysis

Data from the interviews, questionnaires, and documents was analysed manually by means of iterative thematic analysis [37]. The themes corresponded to the Pettigrew and Whipp framework including the content, context, process and outcome of the RG.

The material (i.e., transcribed interviews, questionnaires, and documents) were read through, the aim being to get an overview of the content. Afterwards, the process of coding began, which aimed to identify segments in the data, as well as key concepts or sentences addressing the study’s aims and research questions. In this initial analysis, the coding involved identifying appropriate segments and labelling them with a code that summarized the content. The codes were then transferred from the margins to coding sheets. Next, the codes were compared to identify similarities and differences, and similar codes were sorted into preliminary themes. Afterwards, the emerging themes were reviewed and texts were written to describe the content. The themes were defined and labelled with headings. Each theme described different aspects and patterns in the data. In our results, quotations are used to illustrate the relation between the data and the categorization, as well as to increase the study’s transparency.

The open-ended questions from the questionnaire consisted of short sentences, usually just a few words describing facilitators and potential barriers. These responses were analysed using the same procedure as described above.

Considering the number of documents included in the study (85), the analysis was governed by the content of the documents and divided into four subcategories: information (e.g., information about the RG to patients or clinicians), directive (e.g., criteria for selection of patients), support (e.g. describing how to accomplish MMR), and evaluation (e.g. councils reporting the number of treated patients).

The documents were then analysed as described above. In the latter phase of the analysis, the themes derived from the documents were compared to the themes from the interviews with a focus on similarities and differences. Themes reflecting similar patterns and aspects were compared, and this pattern-matching technique was used to evaluate the trustworthiness of the data and to increase the confidence in our findings.

The data were analysed by EBB, with continuous support from GB. When compiling the analyses of the interviews, questionnaires and documents, the research group continuously discussed the emerging themes and agreed on the final themes [38].

### Ethical considerations

The Regional Ethics Review Board in Stockholm approved the study’s design (Reg. no. 2013/1638-31/1). All counties in Sweden decided to participate in the RG. Once participating, each county agreed to participate in evaluations of the implementation process. Before the interviews, all participants were informed that their participation was voluntary, that they had the right to withdraw at any time without providing any reasons, and that it would not be possible to identify them in the report of the findings. Oral informed consent was obtained from all informants. Completed questionnaires were understood as the participants giving informed consent.

Because the study was an evaluation conducted at the behest of the Ministry of Health and Social Affairs, the participants were informed about the research group’s independent and external role.

## Results

### The implementation process from the national to the clinical level

The RG was rolled out nationwide, targeting the 21 county councils in Sweden, from the national level (i.e., governmental level), to the county councils’ management level (i.e., regional level) and the clinical level within each county council (i.e., local service level). The implementation was confirmed by four governing factors: (1) a financial incentive for county councils to participate in the RG; (2) a political resolution between the Swedish Association of Local Authorities and Regions (representing the county councils in Sweden) and the Swedish Government; (3) guidelines from the relevant authorities; and (4) and evidence-based knowledge forming the basis for what therapies were to be offered within the RG.

### Establishment of project organizations at the county council management level

In all counties, formal project organizations were established at the county council management level with the specific aim of disseminating information concerning the RG, the county’s own aims and goals regarding the RG, and realization of the RG at the healthcare units. These project organizations were either designed for this purpose, or based on previous formal project organizations. These organizations acted in a supportive role, and arranged network meetings within and across the counties to exchange examples of good practice and ways of working. The project organizations produced documents, such as information and directives, for the administrative and clinical level in each county.

The project organizations were placed at each county council’s management level, under the expert guidance of one or at most three process managers. The project organizations also included coordinators and, in the latter phase of the RG, rehabilitation coordinators located at the healthcare units.

Recurrently in the data, the project organizations were described as highly important in leading the implementation process. The organizations seem to have an important assistive and unifying role, facilitating communication between the management, administrative, and clinical levels in the counties, and with other counties and the Swedish Association of Local Authorities and Regions. As expressed in an interview with a first-line manager:

‘Thanks to the coordinators and the process manager, who really have been the driving forces in the implementation. As I see it, I think we have managed to do more than we initially expected.’

### Recruitment and in-service training

To successfully implement the RG’s intentions, from the national level to each county council’s clinical level, recruitment of new staff and in-service training were described as necessary in most of the county councils. At the outset of the RG, the management in several of the counties made an inventory of the healthcare units staffs’ skills and highlighted the need for competence required by the RG. Afterwards, education and in-service training in PT and MMR were offered to the staff at the healthcare units, and recruitment began, if needed. As reported in the interviews, educational efforts were primarily directed to PT included in the RG. Educational efforts were also directed at physiotherapists and occupational therapists concerning MMR (e.g., about mindfulness and relaxation), but to a lesser extent than for PT.

The need for in-service training in PT varied across the counties. Access to expertise in PT at the healthcare units was mainly seen as a result of experiences of similar projects in the counties, i.e., aimed at mental illness. One county had limited access to competence in PT, and as a consequence the starting point of the RG in the county was delayed. As an administrator representing the county said during an interview:

‘Psychosocial competence in primary healthcare has been weak, and is now being built-up gradually. Our county council is behind the other county councils, which have counselors, psychologists and psychosocial teams at the healthcare units.’

### Dissemination of information on the RG from the county council management to clinical level

At each county council management, developed routines for putting the RG’s intentions into practice at the clinical level, addressing what therapies to offer, the content in these therapies, and which professionals should perform the therapies. Advice on how to adapt and establish practices at the clinical level, criteria for inclusion of patients, and the professions required for working with MMR were also communicated in the documents. The findings suggest that documents were adapted to local conditions. As one participant described during an interview:

‘We have based everything in the RG on a routine… a common routine for the county council, in which we recommend how to put the routine into practice, which competencies are needed to perform the recommended therapies, and a kind of systematic quality measurement with questionnaires that the healthcare units have to use’.

Each counties’ documents on the RG, developed at the management level, aimed to direct and support the development of local routines at the clinical level. With respect to the original political resolution, the stated aims of the RG are rather similar in the county documents. However, a shift over time from the main purpose of return-to-work stated in the RG to a focus on care and health could be observed. The required work-related approach was only sporadically mentioned in the documents, exemplified here in a document describing the development of MMR at a healthcare unit, which does not mention return-to-work:

‘The aim of the work has been to, by means of team cooperation, develop the possibilities for rehabilitation among patients with long-term or chronic pain, as a part of the multimodal rehabilitation that is offered patients here at the healthcare units’.

As an effect of the RG, all county councils developed new MMR teams located at the clinical level within the primary healthcare facilities. Team building is a complex process, requiring cooperation across professional boundaries. As stated in interviews with the first-line managers at the healthcare units, most of the staff had worked individually in their professional role, in quite a traditional manner. Among staff with experience of working in MMR teams, the team per se meant deviating from the traditional healthcare hierarchy (where, e.g., the physician or physiotherapist is a biomedical expert who is used to work ‘solo’. Cooperation in teams seemed to contribute to team members’ professional knowledge regarding clinical assessment and treatment of patients, as well as to bringing the individual member’s knowledge to the team and cooperating in decision-making. During an interview, one participant reported:

‘The team develops cooperation around the patient from different professions’ perspectives, and the focus on the patient increases’.

Furthermore, all county councils had, at the clinical level, specialized rehabilitation units working in line with MMR treatment, and therefore from the outset of the RG they had the resources, staff, and teams required for implementing MMR.

### Contextual factors influencing the implementation process

#### Facilitating factors

The financial incentive provided by the national level, involving economic compensation for every completed rehabilitation, was described as facilitating education and recruitment. Experiences from other projects had facilitated access to relevant competence, as mentioned in a questionnaire:

‘Previously, we have purposefully invested in in-service training in cognitive behavioural therapy’.

The Swedish Association of Local Authorities and Regions was mentioned as an important stakeholder in facilitating and developing project organizations at the county council management level. The process managers and first-line managers described establishment and support of project organizations as important, and meant that county council management prioritized implementation of the RG. County council management and managers from healthcare units reported that the project organizations facilitated implementation at the clinical level.

Decisions and routines approved by county council management level defined practices related to the RG as prioritized work. The clinical level – with its qualified and trained first-line managers and staff, along with positive attitudes towards teams and teambuilding - facilitated the implementation, as did previous experience working in line with MMR.

#### Barriers

According to the participants, the RG’s project form, including annual renegotiations, was a hindrance. Delayed decisions from the Swedish Association of Local Authorities and Regions, at the national level concerning, for example, criteria for selection and financial incentives were also seen as obstructing implementation at the county council management level and as resulting in, for example, a shift in inclusion criteria, from the resolution’s time limit of 6–8 weeks of sick leave, to more than 8 weeks.

The national level guidelines concerning one of the core components of MMR did not specify the treatment modalities, and this was reported to have negatively affected the quality of the intervention, as most of the MMR teams, as mentioned before, were new and had been created at the clinical level with currently employed staff primarily used to practicing regular short-term care. Most of the critique was directed at the lack of manuals on how to practice MMR with a strong focus on return-to-work and reduced absence due to illness. Some participants criticized the goal of return-to-work, referring to the inexperience and limited ability of staff at the clinical level to influence patients’ work practices, while others criticized return-to-work for being too diffuse to allow measurement of rehabilitation effects. Lack of well-defined criteria for patient selection (except for absence due to illness before treatment) meant uncertainty as to which patients should be offered MMR. The clinically adapted documents revealed a shift in the time of sick leave, from the RG’s 6–8 weeks up to 12 weeks. This lack of patient selection criteria in the documents, as well as shifting time limit criteria for sick leave were reported to result in uncertainty about which patients to include. As a result, few gainfully employed patients were treated, while those on long-term disability or even those on disability pension were included.

MMR was criticized for being a time- and resource-intensive treatment. Accordingly many first-line managers at the clinical level, who are responsible for economizing hesitated to participate in the RG. As reported in one document:

‘The most common causes for not starting MMR are insufficient number of patients for a group exercise, and/or not having the time needed to deliver this form of more resource-intensive intervention’.

The lack of competence in PT at the clinical level and lack of persons to recruit were seen as barriers to beginning implementation of the core components. Some of the participants, representing county council management and clinical levels, reported negative attitudes to the choice of PTs included in the RG, even though the positive effects of the therapeutic models included were supported by evidence from international research. Introduction of the RG resulted in currently employed psychotherapists without formal training in cognitive behavioural therapy having to be trained and having to refrain from using their competencies in other therapy forms; thus their ‘old’ competence was indirectly disregarded. Furthermore, the county councils recruited new staff trained in these therapies. This meant a delayed start for the RG in some of the county councils, but also an increased risk that staff would not adhere to the new methods in practice. One participant offered the following description during an interview:

‘Within the psychotherapist profession, there are different schools of psychotherapy, and, as we argue, these therapies show similar results. If the therapies hadn’t been limited to cognitive behavioural therapy, we could have used the employed psychotherapists experienced in other therapies, instead of, as we do now, educating psychotherapists in cognitive behavioural therapy’.

The time window for implementing the RG, from each county council’s management level to the clinical level, was seen as too narrow. The dominant opinion expressed in the interviews was that changing organizations and creating new teams are time-consuming, and thus barriers implementation. The working methods used in a work-related approach, to achieve return-to-work, deviated from the traditional methods used in the healthcare services, i.e., treating illness, easing suffering and increasing quality of life. The emphasis on traditional perspectives, found mainly at the county council management and clinical level, was thus a barrier to implementation.

Fragmentation of the previously comprehensive county primary care was described as another potential barrier. This meant patients’ rights to choose their primary care provider, but also the introduction of private care providers as well as free-market competition between providers. Thus, there were no incentives for providers to cooperate at the clinical level. Furthermore, giving priority to severe cases as well as other tasks and treatments in primary healthcare risked diffusing the rehabilitation perspective. In line with this, one participant said during an interview:

‘Within the healthcare system, there are no “quick and easy” solutions. All the time, there are like thirty-nine different ongoing changes and quality improvement interventions, such as national guidelines for stroke. We are expected to work in new and/or different ways, in many areas, such as stroke and diabetes’.

Another barrier described at the clinical level was the high turnover of experienced physicians at the healthcare units, which affected the possibility to work in well-functioning teams. A summary the contextual factors influencing the introduction of the RG are presented in Table 1.

**Table 1:** **Summary of contextual factors influencing the introduction of the rehabilitation guarantee as reported in one or several county councils**

**Contextual factors reported to help the implementation process**	
-	The financial incentive
-	Support from key stakeholders in facilitating and developing project organizations
-	The establishment and support of project organizations
-	Positive attitudes towards teams and teambuilding
-	Previous experiences from working with similar initiatives
**Contextual factors reported to hinder the implementation process**	
-	The project form, including annual renegotiations and narrow time window for implementation
-	Delayed decisions concerning criteria for selection and financial incentives
-	Unspecified treatment modalities
-	Lack of well-defined criteria for patient selection
-	Late introduction of time- and resource-intensive treatment
-	The lack of competence in PT at the clinical level
-	Recruitment difficulties
-	Negative attitudes to the choice of PTs included in the RG
-	Fragmentation of a comprehensive county primary care
-	Turnover of experienced physicians at the health care units hindering the possibility to work in well-functioning teams

### Intermediate outcomes of the RG’s intentions

Establishing project organizations at each county council’s management level was reported to increase the organizations’ readiness, i.e. the county councils’ prerequisites for achieving the RG’s intentions, for future quality improvement efforts.

Another intermediate outcome was the general perception of an increase in competence in cognitive behavioural therapy at the clinical level. Owing to the national compensation system for meeting recruitment and in-service training needs, therapists achieved general competence in cognitive behavioural therapy, which is useful for treatment of mental health problems other than those included in the RG.

The RG involved a process that was reported to entail a shift from reactive to preventive healthcare. The interviewees emphasized that the RG shed light on patient groups and their need for adequate rehabilitation. They felt that early rehabilitation was given more adequate priority after onset of the RG. One document concerning instructions for MMR focussed on the clinicians’ perceptions of the importance of the patient group, and contained the following text:

‘The team has a common value; a biopsychosocial perspective and a holistic view of the patient, which is put into practice during examination and treatment. The patient is seen as a subject’.

## Discussion

To our knowledge, this is the first study to report on implementation of a nationwide rehabilitation programme informed by the Pettigrew and Whipp model of strategic change management [31,32]. Our findings suggest that the establishing of project organization contributed to realization of the RG. In most of the county councils, recruitment and in-service training were described as necessary to fulfilling the RG’s intentions. Our results also show that financial incentive and experience of working in similar projects were beneficial to implementation. Barriers were the short-term project form of the RG, a time window perceived to be too narrow for implementation of the specified methods, clinicians’ attitudes to and competence in working with return-to-work, the lack of manuals and guidelines describing treatment modalities in MMR, and finally the lack of well-defined criteria for patient inclusion. The emphasis on return-to-work in the original presentation of the RG became less pronounced during the implementation process. Instead, care and health were more often described in documents used to disseminate information on the RG. The intermediate outcomes were as follows: patients with rehabilitation needs were given more adequate priority; increased readiness for future implementation efforts; increased general competence in cognitive behavioural therapy and teamwork, which thus became available to patient groups other than those included in the RG.

Consistent with previous research by Wandersman and Florin [39], our findings underline the importance of dedicating time and resources to the implementation process for establishing common goals on all levels (i.e., county council management and clinical level), meeting competence requirements, and for creating structured guidelines for ‘what practices should be done and for whom’ that are firmly established within the organizations.

Following the applied study framework, one group of key findings linked to the content, process, context, and intermediate outcomes of the programme are discussed. The RG’s content introduced evidence-based therapies to professions that had varying evidence-based daily practice. In general, guidelines based on evidence seem to show higher adherence [40]. The evidence-based therapies included in the RG might have contributed to critical reflexion concerning clinical issues and thus increased the status of the professions. However, the RG’s main goal of return-to-work and reduced absence due to illness were criticized by clinicians as being too diffuse to allow measurement of rehabilitation effects in these patients [41]. This finding is in conflict with previous research [40]. This might explain the shift over time from the goal of return-to-work to the general goal of healthcare and preventing illness, hence the less successful realization of return-to-work.

Changing practice requires that changes become part of the prevailing perspective, or social norms in the surrounding context [42]. Concerning the content of the RG, most barriers were reported in relation to MMR, which the clinicians felt was a complex, time- and resource- intensive treatment. In line with our findings, a negative influence on implementation can occur when intervention characteristics and contents are perceived as complex [40]. Initiating interventions from a central authority is known to hinder implementation [43]. However, our results show that specific information on the target patient group and the content of rehabilitation was requested from the county councils, thus local adaptation was a hindrance to implementation. Factors that served as barriers in relation to MMR were highlighted, such as the number of professions included in MMR teams that had to change their practice from working ‘solo’ to engaging in collaborative teamwork [33]. Performing MMR requires qualified and trained staff who can cooperate in teams during clinical assessment and treatment of patients in healthcare units and specialized care modalities. The location of the MMR teams varied across as well as within the counties. This result underlines the importance of addressing contextual factors and current conditions and deviates from previous studies suggesting that adaption to the local context and conditions increases the possibilities for successful implementation [43,44].

Legitimacy through evidence-based procedures was organised through implementation in a two-step, top-down process. Macro-level implementation, i.e., the directives and routines communicated from the Swedish Association of Local Authorities and Regions, was aimed at the micro level, i.e., the clinical level, by the project organizations via process managers. The project organizations were seen as facilitators; establishment of the organizations showed that the RG implementation was prioritized, having support and dissemination functions on the micro-level. Similar findings were reported by Øvretveit et al. [33]. In our study, micro-level implementation was carried out through initiatives from clinical leaders and dedicated staff, underlining the fact that top-down and bottom-up commitment are needed if changes are to succeed [29,45]. Other important facilitators are targeted implementation actions run at the macro-level, such as financial incentives, guidelines and practical recommendations, written material, and workshops [33,40].

Given the knowledge that county councils are extensive, multifaceted organizations, implementation of the RG was likely to face competing agendas [27]. Previous studies have stressed that competing agendas and shifting policies might put implementation on hold [43]. Nevertheless, as shown in our results, experience from participating in similar projects was another contextual factor that positively influenced the organizations’ receptiveness to implementing the RG [45].

Finally, our study reveals the process’s intermediate outcomes. One such outcome is the RG’s impact on clinicians’ attitudes towards the patient group. The interviewees emphasized a focus on patient groups and their need for adequate rehabilitation. In line with these findings, Löfgren et al. [28] showed that implementation of a quality improvement programme increased staff attention to the patient group. Another issue related to the patient group is the possible negative side effects of the implementation for other patient groups, due to the risk that other diagnoses may have been superseded by those included in the RG.

Although MMR may be considered secondary or tertiary prevention, it was perceived as proactive clinical work in contrast to the usual ‘reactive’ work. This might mirror the focus on the individual as an active and responsible agent in MMR rehabilitation and its emphasis on lifestyle, coping strategies, and relapse prevention.

The present study was part of an evaluation commissioned by the Ministry of Health and Social Affairs. However, in the information given to the participants, the research group’s independent role was clearly stated. This was done to minimize the risk of receiving biased information from the participants.

The validity of retrospective data is always a challenge and this study was limited in collecting interview data from a limited number of informants, interviewed about two years after implementation onset. A general reflection is that our evidence does not give high certainty about which actions or context factors were most important in implementing the rehabilitation guarantee, given that most of the data was from interviews and drew on informants’ judgements. Nevertheless, strength of the study was in being able to identify a set of influences by comparing and synthesising judgements from several informants, which likely helped reduce the bias from e.g. memory decay. Repeated interviews might have contributed to a richer process description. However, the questions were designed to support participants’ retrospective and prospective reflections on the RG. At the time of the interviews, the outcomes of the effect evaluation were known to the public. One question in the interview guide addressed these results. This may have influenced participants’ perceptions about the implementation process inasmuch as fewer extreme views were found (both positive and negative) and our informants had a more realistic approach at the time.

The strength of the present study was its use of and ability to integrate data from multiple sources. We used three data sources (questionnaires, interviews, and documents), and our conclusions were based on the consistency of data from these sources. The data were analysed by the research group; the coders represented different theoretical perspectives, scientific backgrounds and methodological orientations (i.e., qualitative and quantitative methods). Hence, interdisciplinary triangulation was applied [46,47] aimed at increasing the rigor of the data collection and analysis process. One limitation of the study is the lack of measurement of the implementation’s fidelity, for example, dose delivered and dose received cf. [48].

## Conclusions

The financial incentive coming from the national level was an overall facilitating factor for implementation at the county council management as well as the clinical level. The RG’s project form and timeframe for implementation and evaluation were perceived as barriers and were reported to hinder the implementation process at the county council management and clinical levels. The model of strategic change applied in the present study helped in identifying factors that influenced the overall implementation process, from the national to the clinical level. Further research might add a cost-effectiveness component, as the cost of an implementation is often related to the magnitude of the programme. Further research might also build on our findings to test more specific relationships between large-scale change implementation processes and intermediate and final outcomes. Our findings suggest several courses of action for policymakers, and we conclude by underlining the importance of dedicating time to and investing of sufficient resources in the activities and in supporting the implementation process at all levels. Needs assessment and description of the local context should precede any implementation process, and dedicated change agents are crucial in the implementation process from the national to the clinical level.
